# CREATING STRENGTH IN AGE: HARNESSING THE POWER OF ARTS AND HUMANITIES NETWORKS

**DOI:** 10.1093/geroni/igz038.110

**Published:** 2019-11-08

**Authors:** Gay P Hanna, Pamela Saunders, Niyati Dhokai

**Affiliations:** 1 Hanna Merrill Inc., Arlington, Viriginia, United States; 2 Georgetown University, Washington, District of Columbia, United States; 3 George Mason University, Fairfax, Virginia, United States

## Abstract

Arts and Humanities networks harness social capital in the service of older populations creating strength in age. This symposium will feature presentations in aging, arts, education, health and humanities exemplifying enormous and often underutilized resources readily available to engage older people across the spectrum of aging to combat decline and frailty at cognitive and physiological levels. Presenters will describe innovative partnership projects such as Sound Health, an initiative developed by the National Institutes of Health and the John F. Kennedy Center for the Performing Arts to expand knowledge and understanding of how listening, performing, or creating music could be harnessed for health and well-being; hybrid arts and humanities in health programs based within medical systems such as the Center for Performing Arts in Medicine at Texas Medical Center: Houston Methodist promoting research/evaluation of arts inventions to improve overall quality of patient care; and, MedStar Georgetown Lombardi Arts and Humanities Program providing a continuum of support for older patients and their caregivers from diagnoses through treatment processes. A Georgetown University case study will be presented on how arts, ethics and humanities are necessary and ideal components of an interdisciplinary master’s degree program in aging studies to ensure understanding a diverse and inter-generational cohort and student’s cultural value systems. The symposium will conclude with a presentation from the National Endowment of the Arts describing program service infrastructures across the country supporting arts engagement of older people, their families and caregivers focusing on lifelong learning; health and well-being; and age friendly design

